# Toxicity of Carbon Nanomaterials and Their Potential Application as Drug Delivery Systems: In Vitro Studies in Caco-2 and MCF-7 Cell Lines

**DOI:** 10.3390/nano10081617

**Published:** 2020-08-18

**Authors:** Rosa Garriga, Tania Herrero-Continente, Miguel Palos, Vicente L. Cebolla, Jesús Osada, Edgar Muñoz, María Jesús Rodríguez-Yoldi

**Affiliations:** 1 Departamento de Química Física, Universidad de Zaragoza, 50009 Zaragoza, Spain; 2 Departamento de Bioquímica y Biología Molecular, Universidad de Zaragoza, 50013 Zaragoza, Spain; taniaherrero1992@gmail.com (T.H.-C.); josada@unizar.es (J.O.); 3 Departamento de Farmacología y Fisiología, Universidad de Zaragoza, 50013 Zaragoza, Spain; mpalosmarg@gmail.com; 4 Instituto de Carboquímica ICB-CSIC, Miguel Luesma Castán 4, 50018 Zaragoza, Spain; vcebolla@icb.csic.es (V.L.C.); edgar@icb.csic.es (E.M.); 5 CIBEROBN (ISCIII), IIS Aragón, IA2, 50009 Zaragoza, Spain

**Keywords:** cytotoxicity, carbon nanomaterials, drug delivery, doxorubicin, camptothecin, Caco-2, MCF-7

## Abstract

Carbon nanomaterials have attracted increasing attention in biomedicine recently to be used as drug nanocarriers suitable for medical treatments, due to their large surface area, high cellular internalization and preferential tumor accumulation, that enable these nanomaterials to transport chemotherapeutic agents preferentially to tumor sites, thereby reducing drug toxic side effects. However, there are widespread concerns on the inherent cytotoxicity of carbon nanomaterials, which remains controversial to this day, with studies demonstrating conflicting results. We investigated here in vitro toxicity of various carbon nanomaterials in human epithelial colorectal adenocarcinoma (Caco-2) cells and human breast adenocarcinoma (MCF-7) cells. Carbon nanohorns (CNH), carbon nanotubes (CNT), carbon nanoplatelets (CNP), graphene oxide (GO), reduced graphene oxide (GO) and nanodiamonds (ND) were systematically compared, using Pluronic F-127 dispersant. Cell viability after carbon nanomaterial treatment followed the order CNP < CNH < RGO < CNT < GO < ND, being the effect more pronounced on the more rapidly dividing Caco-2 cells. CNP produced remarkably high reactive oxygen species (ROS) levels. Furthermore, the potential of these materials as nanocarriers in the field of drug delivery of doxorubicin and camptothecin anticancer drugs was also compared. In all cases the carbon nanomaterial/drug complexes resulted in improved anticancer activity compared to that of the free drug, being the efficiency largely dependent of the carbon nanomaterial hydrophobicity and surface chemistry. These fundamental studies are of paramount importance as screening and risk-to-benefit assessment towards the development of smart carbon nanomaterial-based nanocarriers.

## 1. Introduction

Carbon nanomaterials are promising new materials to be used as drug nanocarriers suitable for medical treatments in biomedicine, due to their large surface area and chemical stability that allows efficient loading of drugs via both covalent and non-covalent interactions [1,2,3]. Although their interaction with lipid membranes and their subsequent intracellular transport is poorly understood, it has been demonstrated that they can enter cells using various endocytic processes [4,5]. A combination of increased tumor vascular permeability and insufficient lymphatic drainage, resulting in what is termed as enhanced permeability and retention (EPR) effect, enables these nanoparticles to transport chemotherapeutic agents preferentially to tumor sites as compared to healthy tissues, thereby reducing toxic side effects [6]. Furthermore, these systems could be used for formulation of hydrophobic molecules which lack of suitable physicochemical characteristics required for development of stable pharmaceutical dosage form. Transition metal contamination from synthesis procedures can be avoided by purification [7,8]. Poor aqueous dispersibility and high aggregation tendency of pristine carbon nanomaterials can be sorted out by appropriate surface functionalization towards their applications as nanocarriers [9]. Functionalization of carbon nanomaterials can be achieved by either non-covalently coatings with amphiphilic macromolecules like lipid, polymers and surfactants, or covalently with hydrophilic functional groups. Specifically, in vivo studies have shown that functionalization with polyethylene glycol (PEG) allows to achieve prolonged circulation half-life, resulting in so-called ‘stealth’ behavior, and therefore improved accumulation in tumor by escaping opsonization-induced reticuloendothelial system (RES) clearance [10,11,12,13], making these nanocarriers good candidates for cancer diagnostics and treatment. Also PEGylated nanomaterials exhibited remarkably reduced in vivo toxicity, avoiding accumulation in liver or spleen.

To further enhance the therapeutic efficacy of drugs and simultaneously diminish their undesirable systemic side effects, different small targeting molecules such as folic acid (FA) [14,15], ligands with strong affinity against a given receptor overexpressed in a tumor [16], antibodies that recognize tumor-associated antigens [17,18,19,20] and also magnetic nanoparticles [3,15] can be further incorporated onto the drug-loaded carbon nanomaterials to confer either active targeting capabilities via receptor-mediated endocytosis or local nanocarrier accumulation induced by external magnetic field. However, addition of excess targeting ligands also increases clearance by the RES because more proteins are now ‘visible’ on the surface than with PEG. Also, adsorption of proteins and other biomolecules could shield the targeting ligands that have been grafted onto the surface of nanocarriers from binding to their receptors on tumors. ‘Stimuli-responsive’ drug delivery, that is, strategies to release drug cargo upon experiencing certain tumor-specific triggers (i.e., higher temperature and lower pH) can be extremely useful for selective and controllable drug release [15,21].

There is a trend to combine the therapeutics and early diagnostics (namely theranostics) together [3,18,22,23,24,25,26]. By combining imaging labels with therapeutics in the same platform, the location of the tumor can be precisely delineated, and the optimal drug doses as well as therapeutic time frame could be determined by acquiring the real-time drug distribution information in vivo. Imaging tags like radioactive nuclides [27,28,29,30] and fluorescence probes [30,31] can be conjugated in carbon multifunctional nanoplatforms to observe their intracellular trafficking and biodistribution in vitro and in vivo. Raman signals from nanocarbon materials can also provide a reliable method to monitor their distribution and metabolism in vivo [32,33].

In addition, carbon nanotubes, carbon nanohorns and reduced graphene demonstrate strong optical absorption in the near-infrared (NIR) region, making them promising materials for use in the photothermal ablation of tumors [25,34,35,36]. Carbon nanomaterials also offer promise in combination therapy, that generally refers to two or more therapeutic agents co-delivered simultaneously, and is becoming more popular because it generates synergistic anticancer effects, enabling a low dosage of each compound and overcoming multi-drug resistance (MDR) cancer [37,38]. While the delivery of cancer chemotherapeutic agents with carbon nanomaterials has been more widely attempted, carbon nanomaterials have also demonstrated promising potentials to be used to deliver many non-anticancer drugs, such as antimicrobial, anti-inflammatory, antihypertensive and anti-oxidant agents [1,26].

However, the biomedical applications of carbon nanomaterials arouse serious concerns, as more information on the pharmacokinetics, metabolism, long-term fate and toxicity is essential [27,29,31,39,40,41]. The issues of toxicity surrounding the biomedical applications of carbon nanomaterials still remain controversial to this date, with studies demonstrating conflicting results [42,43,44,45]. Carbon nanomaterial-based drug delivery systems are still considered far from being accepted for use in actual clinical settings. Progress towards clinical trials will depend on the outcomes of efficacy and toxicology studies, which will provide the necessary risk-to-benefit assessments for carbon-based materials. Fundamental studies regarding the impact of size, shape, aggregation degree and functional groups of carbon nanomaterials are needed to provide the design criteria for successful nanomaterial-based strategies. In carbon nanomaterial safety assessment, in vitro cytotoxicity tests are an important research subject because they are fast, reproducible and easy to control the consistency of experimental conditions and should complement and/or supplement in vivo-animal tests. In vitro studies are able to provide information on the biological fate of nanomaterials at the cellular or multicellular levels. In the literature, there is a lack of comparative studies, as extensive variations in the nanomaterial source, functionalization, and experimental conditions do not allow direct comparison of the different results.

We here investigated in vitro toxicity of various Pluronic (F-127)-dispersed carbon nanomaterials in human epithelial colorectal adenocarcinoma (Caco-2) and human breast adenocarcinoma (MCF-7) cell lines. Representative examples, with different size, shape and functional groups on the surface, such as carbon nanohorns (CNH), carbon nanotubes (CNT), graphene nanoplatelets (CNP), graphene oxide (GO), reduced graphene oxide (RGO) and carbon nanodiamonds (ND) were systematically compared under the same experimental conditions.

Furthermore, their potential in the field of drug delivery of anticancer drugs, such as doxorubicin (DOX) and camptothecin (CPT), was also compared in this work. DOX and CPT have been chosen here as examples of hydrophilic and hydrophobic drugs, respectively. DOX belongs to anthracyclines, topoisomerase II inhibitors exhibiting multiple mechanisms of action and high clinical effectiveness against many types of cancer. Notably, cardiotoxicity is a major concern during therapy as it may be dose-limiting [46]. More effective and safer ways of delivering anthracyclines are hence of significant research interest. Also, resistance to anthracyclines and other chemotherapeutics due to P-glycoprotein (P-gp), a membrane transporter that actively pumps doxorubicin out of the cell, is a frequent problem in cancer treatment [47]. It has been reported that nanocarriers enhance doxorubicin uptake in drug-resistant cancer cells [48,49,50]. Thus, DOX enters the cells attached to nanocomposites bypassing the P-gp transporter, detaches from the nanocomposite surface following natural acidification of endosomes, and migrates reaching the cell nucleus. On the other hand, CPT is a potent anticancer agent with topoisomerase I-inhibiting activity. However, its practical use in viable cancer therapeutic systems is greatly hampered due to its low solubility in aqueous media [51]. The need to formulate water-soluble salts of CPT (that is, alkaline solutions for intravenous injections) led to chemical modifications of the molecule with loss of anti-tumor activity [52,53]. Thus, developing new drug delivery nanocarriers for CPT able to transport and deliver the drug inside the cancer cells has recently received considerable attention [54].

Finally, it has to be noted that the cancer cell lines targeted here, Caco-2 and MCF-7, correspond to cancers among those having the highest incidence in Western countries, hence the interest of anticancer therapeutic studies performed on them.

## 2. Materials and Methods

### 2.1. Carbon Nanomaterials

CNT used here are short (average length < 1 µm) and purified (95% wt. %) multi-walled carbon nanotubes from Nanocyl S.A. (Sambreville, Belgium), NANOCYL^®^ NC3150™, produced via catalytic chemical vapor deposition (CCVD) process. CNH were single wall carbon nanohorns provided by Carbonium Srl (Padua, Italy), produced without any catalyst, by rapid condensation of small carbon clusters (C_2_ and C_3_) resulting from direct vaporization of graphite [55]). Single layer graphene oxide (GO, purity 99 wt. %) was supplied by Cheap Tubes Inc. (Grafton, VT, USA). RGO was from Sigma-Aldrich (777684, Darmstadt, Germany). CNP (purity 91 at.%.) and detonation nanodiamonds (ND, purified/grade G01) were purchased from PlasmaChem GmbH (Berlin, Germany).

### 2.2. Characterization of Carbon Nanomaterials

Transmission electron microscopes (TEM, Tecnai T20 and Tecnai F30, FEI, Hillsboro, OR, USA, operating at 200 and 300 KV, respectively) were used to characterize the structural features of carbon nanomaterials. During sample preparation, nanomaterials were dispersed in ethanol, and a drop was placed onto carbon coated copper grids, the sample excess was wicked away by means of a Kimwipe and allowed to dry under ambient conditions. Prior to TEM imaging, the samples on the grids were placed in a O_2_-Ar (20% O_2_) plasma cleaner (Model 1020 Fischione, Hanau, Germany) for 5–10 s to remove organic (hydrocarbon) contamination.

X-ray photoelectron spectroscopy (XPS) was performed on powder samples deposited onto double-sided carbon tape using an ESCA Plus spectrometer (Omicron, Taunusstein, Germany) provided with a Mg anode (1253.6 eV) working at 225 W (15 mA, 15 kV). CasaXPS software (version 2.3.15, accessed on 1 June 2020) was used for the peak deconvolution and Shirley type baseline correction was applied.

Nitrogen adsorption-desorption isotherms were measured at 77 K (Micromeritics ASAP 2020, Micromeritics Instrument Corp., Norcross, GA, USA) and surface area measurements of the powder samples were obtained using the Brunauer–Emmett–Teller (BET) method at values of relative pressure (p/p_0_) between 0.05 and 0.3.

### 2.3. Dispersion of Carbon Nanomaterials

Pluronic^®^ F-127 (F-127), suitable for cell culture, average molecular weight 12.6 µDa, was purchased from Sigma.Aldrich (Darmstadt, Germany). F-127 solutions at 15 µg·mL^−^^1^ and 10 min bath sonication (100 W Branson 2510 bath sonicator, Branson Ultrasonics, Danbury, CT, USA) were used here to assist in carbon nanomaterials dispersion at 3.0 and 0.6 µg·mL^−^^1^ concentration in cell culture medium.

Dispersions of carbon nanomaterials were prepared in cell culture medium without fetal bovine serum (FBS), as it is known that bovine serum albumin (BSA) has different affinity towards carbon nanomaterials. Thus, it has been reported that BSA readily adsorbed on GO, resulting in a decrease in GO toxicity. In contrast, BSA loading capacity was ∼9-fold lower for MWCNT [56].

DOX and CPT loading on carbon nanomaterials was performed by simply mixing of solutions in cell culture media, agitated by using a vortex mixer and kept overnight in dark at room temperature. Due to its poor solubility in aqueous media, CPT was initially dissolved in dimethyl sulfoxide (DMSO, ≥99.9%, from Sigma-Aldrich, Darmstadt, Germany) to a concentration of 1.6 mg·mL^−^^1^, and then diluted on cell culture medium to the required working concentrations.

### 2.4. Cell Lines and Cell Culture

Human Caco-2 cell line (TC7 clone) was kindly provided by Edith Brot-Laroche (Université Pierre et Marie Curie-Paris 6, UMR S 872, Les Cordeliers, France). Caco-2 cells (passages 40–60) were cultured in Dulbecco’s Modified Eagles medium (DMEM) (Gibco Invitrogen, Paisley, UK) supplemented with 20% fetal bovine serum (FBS), 1% non-essential amino acids, 1% penicillin (1000 U/mL), 1% streptomycin (1000 µg/mL) and 1% amphotericin (250 U/mL), at 37 °C under a humidified atmosphere with 5% CO_2_. The cells were passaged enzymatically with 0.25% trypsin-1 mM EDTA and sub-cultured on 25 cm^2^ plastic flasks at a density of 5 × 10^5^ cells/cm^2^. Culture medium was replaced every 3 days. Experiments were performed in undifferentiated cells (24 h post-seeding to prevent cell differentiation).

Human breast adenocarcinoma MCF-7 cells were kindly provided by Carlos J. Ciudad and Dr. Verónica Noé (Departamento de Bioquímica y Fisiología, Facultad de Farmacia, Universidad de Barcelona, Spain). MCF-7 cells were maintained in the same conditions as described for Caco-2 cell line.

For comparison purposes, some experiments were performed with human dermal fibroblasts that were kindly provided by Dr. Julio Montoya (Departamento de Bioquímica y Biología Molecular, Facultad de Veterinaria, Universidad de Zaragoza, 50013 Zaragoza, Spain).

### 2.5. Cell Viability Assay

24 h after seeding in 96-well plates at a density of 4 × 10^3^ cells/well, cells were treated for 24 and 72 h with carbon nanomaterial dispersions (including DOX and CPT anticancer drugs in some studies), and then 3-[4,5-dimethylthiazol-2-yl]-2,5-diphenyltetrazolium bromide (MTT) assay was performed for assessing cell metabolic activity. In short, 10 μL of MTT (5 mg·mL^−1^) were added to each 100 µL sample well and incubated for 2 h. Mitochondrial dehydrogenases of viable cells reduce the yellowish water-soluble MTT to water-insoluble formazan crystals, which are later resolubilized by replacement of the medium with DMSO, obtaining a purple colored solution. Absorbance at 540/620 nm was measured using a SPECTROstar Nano microplate reader (BMG Labtech, Ortenberg, Germany). Control values (sample wells without treatment) were set at 100% viable and all values were expressed as a percentage of the control. All experiments were performed in triplicate. In each of the three independent experiments, each sample result corresponds to 16 wells, which sums up 48 wells per sample.

### 2.6. Reactive Oxygen Species (ROS) Assay

The reactive oxygen species (ROS) production was assayed by the 2′,7′-dichlorofluorescein diacetate (H_2_DCFDA) molecular probe [57,58]. The cell-permeable H_2_DCFDA diffuses into cells and is deacetylated by cellular esterases to form 2′,7′-dichlorodihydrofluorescein (H_2_DCF). In the presence of ROS, H_2_DCF is rapidly oxidized to 2′,7′-dichlorofluorescein (DCF), which is highly fluorescent. Caco-2 and MCF-7 cells were seeded in 96-well plates at a density of 4 × 10^3^ cells/well, incubated 24 h under standard cell culture conditions and then treated with nanomaterial dispersions (3 µg·mL^−1^) for 24 h. Subsequently, cells were washed twice with PBS and incubated for 20 min with 100 μL of 20 μM H_2_DCFDA at 37 °C for in the dark. Fluorescence intensity (ex = 485/em = 535 nm) was measured with FLUOstar Omega microplate reader (BMG Labtech). % ROS production was compared to a negative control (untreated cells) and was normalized with MTT assays at 24 h incubation. All experiments were performed in triplicate. In each of the three independent experiments, each sample result corresponds to 16 wells, which sums up 48 wells per sample.

### 2.7. Cell Death Study

Caco-2 and MCF-7 cells were plated in 75 cm^2^ flasks at a density of 5 × 10^5^ cells per flask and incubated 24 h under standard cell culture conditions. They were then exposed to dispersions of the tested carbon nanomaterials (3 µg·mL^−1^) for 72 h. Each sample result corresponds to a pool of two 75 cm^2^ flasks. Quantitative flow cytometry (FCT) analysis was performed using propidium iodide (PI) intake and FITC annexin V staining according to manufacturer’s instruction. Briefly, cells were washed twice with phosphate saline buffer (PBS) and 100 µL of annexing V-binding buffer (10 mM HEPES/NaOH pH 7.4, 140 mM NaCl, 2.5 mM CaCl_2_) were transferred to a 5 mL culture tube. Additions of 5 µL FITC annexin and 5 µL PI were made to each tube and then incubated for 15 min in the absence of light at room temperature. Cells were then resuspended in 400 μL of annexin V-binding buffer and analyzed with BD FACSAria flow cytometer (BD FACSDIVA version 7.0 software, accessed on 1 June 2020). Untreated cells were used as negative control and the positive control corresponds to cells treated with CPT (0.8 µg·mL^−1^). Preliminary gating was used in flow cytometry analysis to identify the cells of interest based on the relative size and complexity of the cells, while removing debris and other events that are not of interest.

### 2.8. Cell Cycle Assay

Caco-2 and MCF-7 cells were plated in 75 cm^2^ flasks at a density of 5 × 10^5^ cells per flask and incubated 24 h under standard cell culture conditions. Each sample result corresponds to a pool of two 75 cm^2^ flasks. They were exposed to carbon nanomaterial dispersions (3 µg·mL^−1^) for 72 h and then washed with PBS, collected and fixed for 30 min at 4 °C and incubated with 70–80% ice-cold ethanol at −20 °C for 24 h. After washing with PBS and 5 min centrifugation at 2500× *g* rpm, cells were resuspended in PI/RNase staining buffer. PI-stained cells were analyzed for DNA content with a BD FACSArray bioanalyzer. PI fluorescence was measured in the orange range of the spectrum using a 562–588 nm band pass filter, and cell distribution was displayed on a linear scale. The percentage of cells on each cell cycle phase was determined by means of BD ModFit LT version 3.3 software (accessed on 1 June 2020).

### 2.9. Statistical Analysis

The experimental data were analyzed by one-way analysis of variance (ANOVA) followed by Bonferroni post-test using GraphPad Prism software (version 5.02, GraphPad Software, Inc., San Diego, CA, USA, accessed on 1 June 2020). Interval plots display 95% confidence intervals for the mean. Data were presented as means  ±  S.D. and differences were considered significant at *p* < 0.05.

## 3. Results

### 3.1. Characterization of Carbon Nanomaterials

Frequently, the most likely source of the apparent lack of uniformity in the results reported in the literature for in vitro and in vivo studies is the different structural and physicochemical properties of the diverse nanomaterials used. Thus, there are huge dissimilarities (i.e., length, diameter, surface defects, oxygen content, presence of impurities, etc.) among the batches employed by researchers. Therefore, thorough characterization studies of the carbon nanomaterials are required and must be taken into consideration to obtain meaningful results.

#### 3.1.1. Transmission Electron Microscopy (TEM)

**Figure 1:**
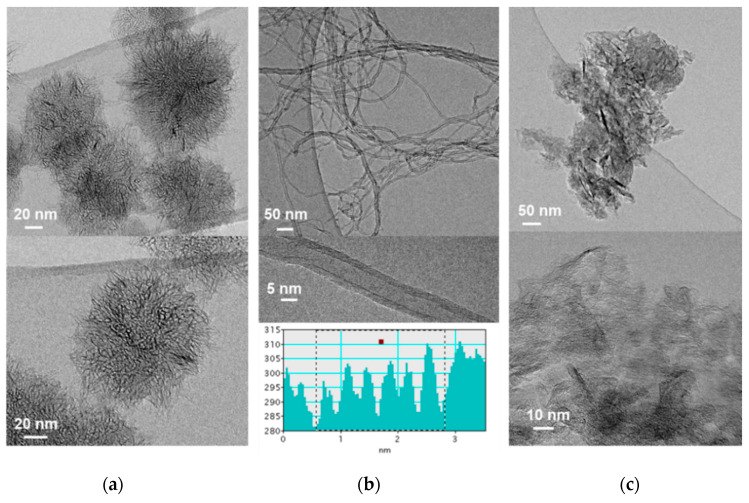

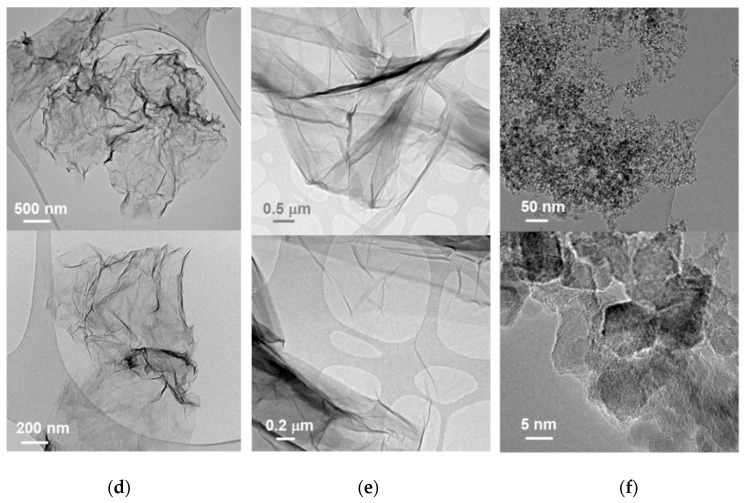
TEM micrographs of the tested carbon nanomaterials: (**a**) CNH, (**b**) CNT, (**c**) CNP, (**d**) RGO, (**e**) GO and (**f**) ND. Inset in (**b**) shows image analysis of a MWCNT with six concentric nanotubes.

The characterization of the structural features and textural properties of the carbon nanomaterials tested here provides useful information on their interaction with drugs and cells. CNH are conical-shaped single-walled tubules that arrange into 100 nm dahlia-like assemblies (Figure 1a). The CNT used here are relatively short MWCNT (up to 1 micron in length) and ~10 nm in diameter, comprising around six concentric nanotubes (Figure 1b). TEM micrographs of two-dimensional, graphene derivatives GO and RGO (Figure 1d,e, respectively) reveal that most flakes are up to 1 micron in length as well as their high exfoliation degree. On the contrary, CNP consist of aggregates of smaller, less exfoliated graphene sheets (Figure 1c). Finally, Figure 1f shows aggregates comprising ND of about 5 nm in diameter.

#### 3.1.2. Photoelectron Spectroscopy (XPS)

X-ray photoelectron spectroscopy (XPS) provides important hints of the surface chemistry of the tested carbon nanomaterials (Figure S1). The ratio of oxygen and carbon atoms was calculated from the O1s and C1s peaks, and the results of the quantitative surface analysis are summarized in Table 1. XPS spectra of CNH, CNT, CNP and RGO are quite similar and correspond to C sp^2^-based nanomaterials materials, with a low O:C ratio and, therefore, are highly hydrophobic. In contrast, GO has a significant high oxygen content (49.2 at.%), as it contains abundant oxygen-containing functional groups, which provide enhanced hydrophilicity. Although O content in ND is not as high as in GO, ND are known to disperse easily in polar solvents, as it will be commented later in the Discussion section. No significant transition metal contamination was observed in XPS spectra.

#### 3.1.3. Specific Surface Area

Specific surface area for carbon nanomaterials in powder, determined using N_2_ adsorption and BET method, are shown in Table 2.

The largest specific surface area value corresponds to CNP. While these values correspond to powder samples, sonication-assisted dispersion in solution significantly increases surface area, which is particularly relevant when it comes to GO exfoliation.

### 3.2. Dispersion of Carbon Nanomaterials

For efficient cellular uptake of carbon nanomaterials, it is necessary that that they remain dispersed and not aggregate in culture medium. Non-ionic polyether surfactants, such as poloxamer triblock copolymers (known also by the trade name Pluronic^®^), are frequently used as dispersants to prepare various nanoparticle suspensions, especially with hydrophobic nanoparticles, such as CNT and related materials. Pluronics are amphiphilic molecules that comprise two polyethylene glycol (PEG) blocks and one polypropylene glycol (PPG) block of various sizes and are frequently used for in vitro and in vivo nanotoxicity studies because they are considered non-toxic dispersants. Thus, the US Food and Drug Administration (FDA) has approved various Pluronic polymers for pharmaceutical usage and even intravenous administration [59,60]. However, it is known that Pluronics can be degraded during sonication, depending on sonication time, power, and frequency conditions, as the collapse of cavitation bubbles generated during sonication can create sufficient heat, pressures, and shear forces to degrade polymers containing PEG, PPG or both. It is therefore important to assess whether sonication of dispersants themselves contribute to the toxicity of sonicated nanomaterial suspensions so as not to misinterpret toxicity results [61]. Figure S2 shows that F-127 decreased MCF-7 and Caco-2 cell viability at high concentration. Thus, F-127 at low concentration (15 µg·mL^−1^) and short bath sonication time (<10 min) was used here to assist in carbon nanomaterials dispersion in cell culture medium, while avoiding the generation of toxic degradation products. Moreover, it is well documented in the literature that above critical micelle concentration (CMC), Pluronics form nano-sized micellar structures which can act as drug nanocarriers, showing higher anticancer activity as compared to free drug [59,62]. It was also checked here that neither DOX nor CPT anticancer activity was enhanced due to drug encapsulation in F-127 micellar structures at the low F-127 concentration used here (Figure S3). Therefore, any improvement achieved in this study in cell killing ability over free drug against cancer cells can be attributed to the drug-nanocarrier complex.

### 3.3. Carbon Nanomaterials Toxicity Assessment

Cell viability assay, apoptosis detection, cell cycle analysis and ROS production assay are useful in vitro methods for the assessment of toxicity of nanomaterials.

#### 3.3.1. Cell Viability Assay

**Figure 2:**
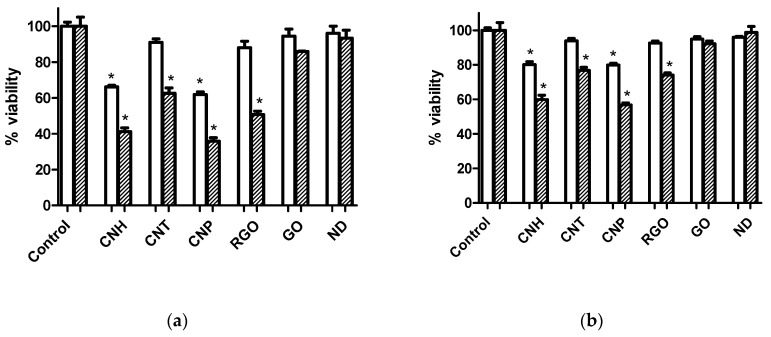
Cell viability assays after 24 h (white) and 72 h (striped) of incubation with various carbon nanomaterials at 3 µg·mL^−1^ showing differential effects on (**a**) Caco-2 and (**b**) MCF-7 cells. Values that are significantly different from the control (*p* < 0.05) are denoted with asterisk (*). Untreated cells were used as control.

Figure 2 shows cell viability assays on carbon nanomaterial treatment at 3 µg·mL^−1^ after 24 and 72 h for Caco-2 and MCF-7 cell lines. Also results at 0.6 µg·mL^−1^ can be found in Figure S4. The MTT assays showed dose-dependence on both Caco-2 and MCF-7 cell lines. Cell viability followed the order of CNP < CNH < RGO < CNT < GO < ND. The decrease in cell viability was more pronounced for the Caco-2 cell line. No significant cell viability decrease was observed in Figure 2 for GO and ND at 3 µg·mL^−1^ (and also for CNT, at 0.6 μg·mL^−1^, as shown in Figure S4).

Interestingly, MTT studies on human dermal fibroblast cells (Figure S5), as example of healthy cells, provided the same viability sequence after carbon nanomaterial treatment, showing less effects than on Caco-2 cells and very similar to those on MCF-7 cells.

#### 3.3.2. Reactive Oxygen Species (ROS) Assays

**Figure 3:**
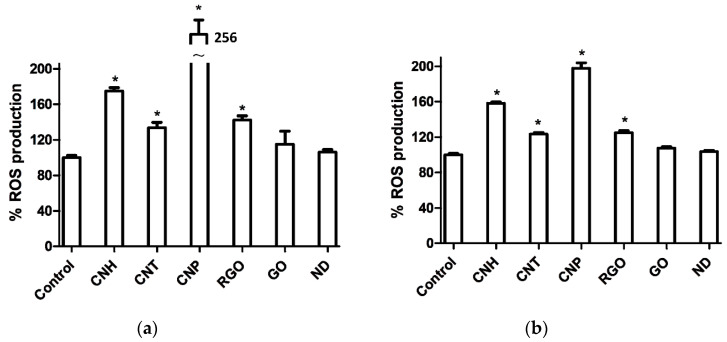
ROS generation on (**a**) Caco-2 and (**b**) MCF-7 cells upon incubation with nanomaterial dispersions (3 µg·mL^−1^) for 24 h. Significant results as compared to untreated control cells are marked by asterisk * for *p*-value < 0.05.

According to Figure 3, CNP produced the highest levels of ROS, more noticeably for Caco-2 cells.

#### 3.3.3. Cell Death Study

**Figure 4:**
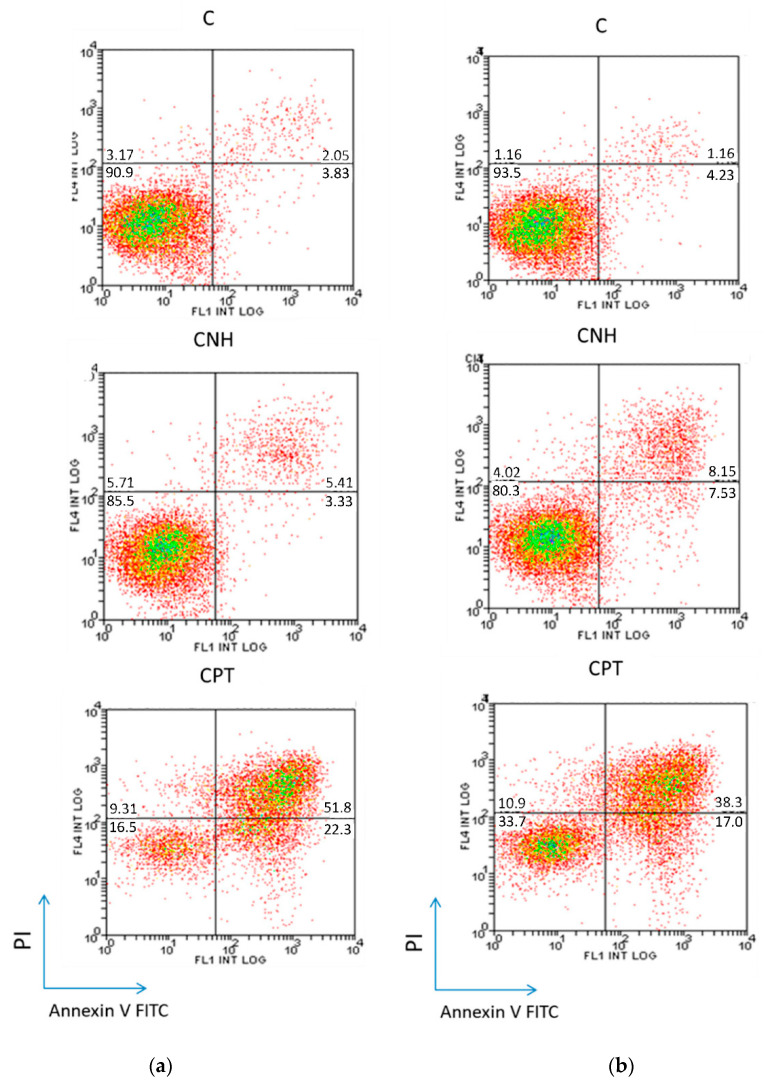
Annexin V/Propidium iodide assay, providing quantitative information about living (lower left quadrant), early apoptotic (lower right quadrant) and late apoptotic and necrotic (upper right quadrant) cells for (**a**) Caco-2 and (**b**) MCF-7 cell lines, treated with CNH at 3 µg·mL^−1^ for 72 h. Data are presented as percentage of the cell population. Untreated cells, denoted as C, were used as negative control, and CPT (0.8 µg·mL^−1^) treated cells were used as positive control.

Figure 4 shows quantitative flow cytometry analyses for the Caco-2 and MCF-7 cell lines, treated 72 h with CNH at 3 µg·mL^−1^, which showed the highest values of late apoptosis/necrosis among the carbon nanomaterials tested here. Experiments were performed as described in Section 2.7, and interpreted as follows: the percentage of viable cells is shown in the lower left quadrant (annexin V^−^/PI^−^), of early apoptotic cells in the lower right quadrant (annexin V^+^/PI^−^), and of late apoptotic and necrotic cells in the upper right quadrant (annexin V^+^/PI^+^). Additional results for CNT, CNP and RGO are compared in Figure S6.

#### 3.3.4. Cell Cycle Analysis

**Figure 5:**
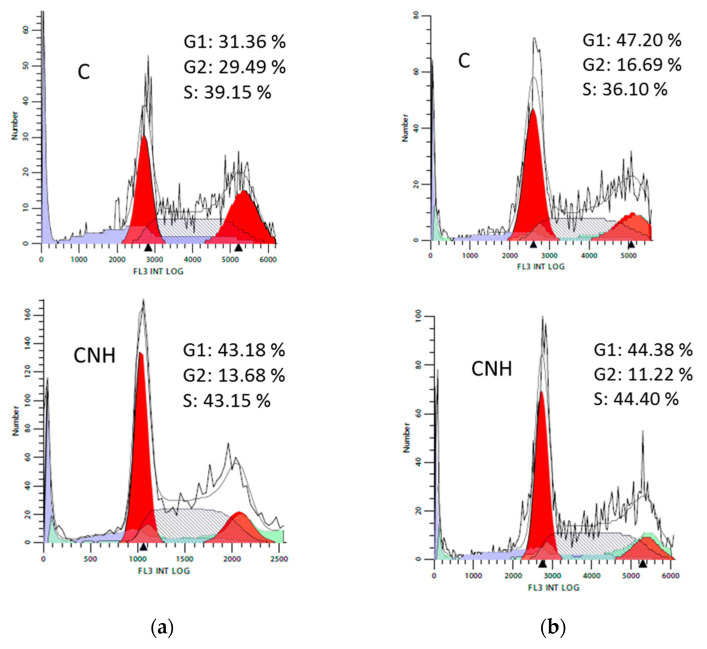
Flow cytometric analysis of (**a**) Caco-2 and (**b**) MCF-7 cell cycle after treatment with CNH at 3 µg·mL^−1^ for 72 h. C denotes untreated control cells.

Figure 5 shows flow cytometric analysis of Caco-2 and MCF-7 cell cycle after treatment with CNH at 3 µg·mL^−1^ for 72 h. Additional results for CNT, CNP and RGO are compared in Figure S7.

### 3.4. Carbon Nanomaterials as Anticancer Drug Nanocarriers

**Figure 6:**
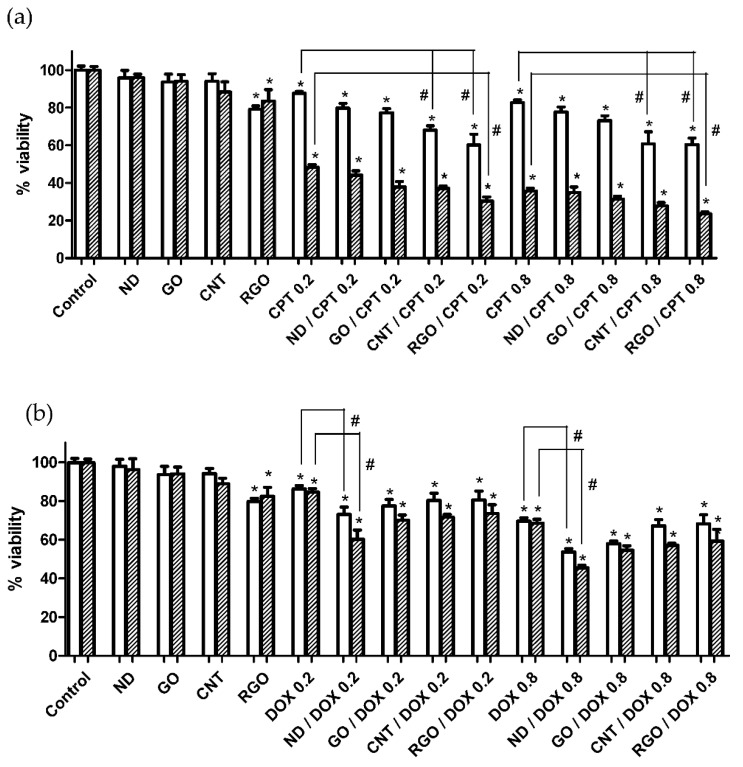
Cell viability assays after 24 h (white) and 72 h (striped) of incubation of Caco-2 cells with ND, GO, CNT and RGO at 0.6 µg·mL^−1^ concentration, free drug CPT (**a**) and DOX (**b**) at both 0.2 and 0.8 µg·mL^−1^ concentrations, and CPT- (**a**) and DOX- (**b**) loaded carbon nanomaterials. (* and # represent significance at *p* < 0.05 when compared to untreated control cells and free drug-treated cells, respectively).

The potential in the field of drug delivery as nanocarriers of anticancer drugs of the four carbon nanomaterials studied here that showed the lowest effect on the cells (ND, GO, CNT and RGO) was compared in Figure 6. MTT assays were performed on Caco-2 cells at two drug concentrations, 0.2 and 0.8 µg·mL^−1^. Carbon nanomaterial concentration was chosen as low as 0.6 µg·mL^−1^, so that the observed decrease in cell viability could be attributable to the improved DOX or CPT efficacy when loaded on carbon nanomaterial nanocarriers rather than to any inherent toxicity of carbon nanomaterials. CPT showed more potent cytotoxic activity than DOX against both cancer cells (Figure 6). Carbon nanomaterial/drug complexes resulted in improved anticancer activity compared to that of the free drug. For CPT, the improvement follows the sequence ND < GO < CNT < RGO. For DOX, the sequence is the opposite (Figure 6). Thus, CNT and RGO showed significant enhanced anticancer activity compared to the free drug, but ND showed a significant improvement when it comes to DOX.

## 4. Discussion

CNT are a type of hollow one-dimensional (1D) carbon-based nanomaterial consisting of a graphene sheet rolled up to form a cylindrical structure with sp^2^ hybridized carbon atoms. CNT are classified into single-walled carbon nanotubes (SWCNT) and multi-walled carbon nanotubes (MWCNT), have high aspect ratios and needle-like shapes [63]. Comparing the two types, there has been a major debate over whether SWCNTs or MWCNTs generate more toxicity. Some research groups have reported that SWCNT cause more apoptosis than MWCNT, as they are more agglomerated [64,65,66]. Moreover, short CNT were found to be less toxic than longer CNT, which is comparable with the observed toxicity of asbestos [44,65,67,68]. CNT used here are MWCNT, with relatively short length (mean length 1 μm, Figure 1b), so that low toxicity is expected. The purity of this CNT material is high (>95.0%) so no significant toxicity should result from any traces of the transition metal nanoparticles used during CNT production.

Single-walled carbon nanohorns (SWCNH) are horn-shaped single-walled tubules with cone angles of approximately 20° that usually form aggregates with diameters of 80–100 nm [69,70] with a “dahlia-like” shape, as shown in Figure 1a. They are produced essentially metal-free and with high purity [71]. Their use in biomedical applications is still at a preliminary stage. SWCNH used here were produced without any catalyst by direct vaporization of graphite, as described in Section 2.1.

Another category of carbon nanomaterial is graphene, a two-dimensional (2D) sp^2^-bonded carbon sheet in a honeycomb structure and therefore, pristine graphene is hydrophobic in nature. On the contrary, GO contains abundant epoxy and hydroxyl functional groups attached to the basal plane and carboxylic groups attached to the edges, that disrupt the π conjugation, providing enhanced hydrophilicity which even enables the efficient dispersion in aqueous media. π conjugation and therefore hydrophobicity are partially restored upon reduction in RGO [72,73]. Size and morphological characteristics of graphene derivatives studied here, CNP, RGO and GO, are shown in Figure 1c–e.

As another important member of carbon nanomaterial family, ND consist of a highly ordered diamond core covered by a layer of functional groups on the surface, such as carboxyl, lactone, hydroxy and ketone, which stabilizes the particle by terminating the dangling bonds [74,75]. ND produced by detonation method are extremely tiny particles with average diameter between 4–6 nm (Figure 1f). ND are becoming increasingly useful in therapeutic and diagnostic applications due to their biocompatibility, scalability, and easy surface modification [76,77].

According to the XPS results summarized in Table 1, CNH, CNT, CNP and RGO nanomaterials have a low O:C ratio and can be considered as hydrophobic and difficult to disperse in polar solvents. On the contrary, GO has a remarkable high oxygen content and can be considered hydrophilic. Although oxygen content in ND is lower than in GO (Table 1), ND are known to disperse easily in polar solvents, which is due to the hydrophilic functional groups on the outer shell. No significant transition metal contamination for the tested nanomaterials was observed by TEM and XPS. Thus, we can claim that the toxicological effects of metal impurities in these nanomaterials are negligible.

Amphiphilic F-127 was used here to assist the dispersion of carbon nanomaterials in cell culture media through noncovalent functionalization, which involves the coating of the carbon nanomaterials with hydrophobic PPG motifs anchored onto the material surface, with the hydrophilic PEG ends extending to the aqueous solution and enabling the stability of the material in aqueous media.

Results of MTT assays upon treatment with carbon nanomaterials at 3 µg·mL^−1^ (Figure 2) and 0.6 µg·mL^−1^ (Figure S4) show that the cell viability was cellular type, time and dose-dependent. Viability decrease was more pronounced on the highly active metabolically Caco-2 cells. Cell viability follows the order CNP < CNH < RGO < CNT < GO < ND for both Caco-2 and MCF-7 line cells. The sequence in cell viability that resulted from the MTT assays for the different carbon nanomaterials tested here can be explained taking into account the surface chemistry of carbon nanomaterials. Thus, oxygen functional groups on the surface of carbon nanomaterials shield the hydrophobic domains. Two groups of carbon nanomaterials can be distinguished here, the hydrophilic ones, ND and GO, which present low effect on cells, and the hydrophobic ones, CNT, RGO, CNH and CNP, which inhibited cell viability in more extent. The highest viability values correspond to ND, whose surface is rich in functional groups, which make them ideal nanocarriers for building drug delivery systems. However, as it will later be discussed, ND efficiently load hydrophilic drugs, such as DOX, which readily attach to their functional groups on their surface, rather than hydrophobic drugs, such as CPT.

Figure 3 shows that, compared to the other carbon nanomaterials, CNP produced the highest ROS levels, more pronounced for Caco-2 cells. We also found enhancement of ROS levels for cells treated with CNH respect to those treated with CNT and RGO. No significant ROS level alterations were however observed for ND and GO.

As for the apoptosis study, the combination of annexin V and PI has been used to discriminate early apoptotic cells from late apoptotic and necrotic ones. Results collected in Figure 4 and Figure S6 show that the hydrophobic carbon nanomaterials induced late apoptosis/necrosis for both Caco-2 and MCF-7 cells, being more pronounced for CNH. Anticancer drug CPT at 0.8 µg·mL^−1^, whose toxicity was much larger than that of all carbon nanomaterials studied here, was used as positive control.

The effect of carbon nanomaterials on cell cycle progression in Caco-2 and MCF-7 cells is shown in Figure 5 and Figure S7. Cytometric analysis showed no significant differences in the percentage of cells in the individual phases of the cell cycle for all the tested carbon nanomaterials and untreated cells, particularly for MCF-7 cells

Taking these results all together, we conclude that ND and GO show low toxicity, which is due to the oxygenated functional groups on their surface that shield the hydrophobic domains. On the other hand, CNH and CNP induce Caco-2 and MCF-7 late apoptosis/necrosis and enhanced ROS levels, which could be associated with the higher decrease in cell viability, compared to other hydrophobic carbon nanomaterials such as CNT and RGO. This could probably be due to the “dahlia-like” CNH morphology, consisting of small structures containing sharp conical ends, that may produce damage to cells, as well as the sharp edges of the highly fragmented CNP platelets [78,79]. Finally, CNP were found to induce the most elevated levels of ROS, which would contribute to the highest observed decrease in cell viability. The effect is noticeably more pronounced on the more rapidly dividing Caco-2 cells.

It is worth noting that CNH was reported to inhibit proliferation of human liver cell lines and promoted apoptosis [80]. In contrast, other authors reported low toxicity for CNH [69,81,82]. It has to be noted that low toxicity reports correspond to CNH synthesis methods leading to oxidized CNH, such as CO_2_ laser ablation or arc discharge. Thus, oxygen functional groups on the surface would shield hydrophobic carbon domains from interactions with cellular membranes. CNH studied here were produced by direct vaporization of graphite, with very low O content, which was confirmed by XPS (Table 1). This highlights the importance of carbon nanomaterial source when drawing meaningful conclusions from toxicity studies.

Finally, the potential of carbon nanomaterials materials in the field of drug delivery of anticancer drug was compared here. Drug delivery systems based on noncovalent interactions have several advantages compared with covalent conjugation. Thus, extra steps required in chemical conjugations are not necessary. Also, because the drug structure is not chemically altered, drug molecules released from such delivery systems are expected to exert their predicted pharmacological effects. Many clinically used chemical drugs possess aromatic rings, such as DOX, playing the π–π stacking interactions the major role in drug delivery systems [83]. It is known that the loading efficiency for DOX decreases when using CNTs with higher levels of PEGylation, due to the increased hydrophilicity of the surface. Furthermore, faster release rates of DOX were observed for these higher PEGylated CNTs owing to the lower affinity of DOX to the PEGylated CNT [84].

**Figure 7:**
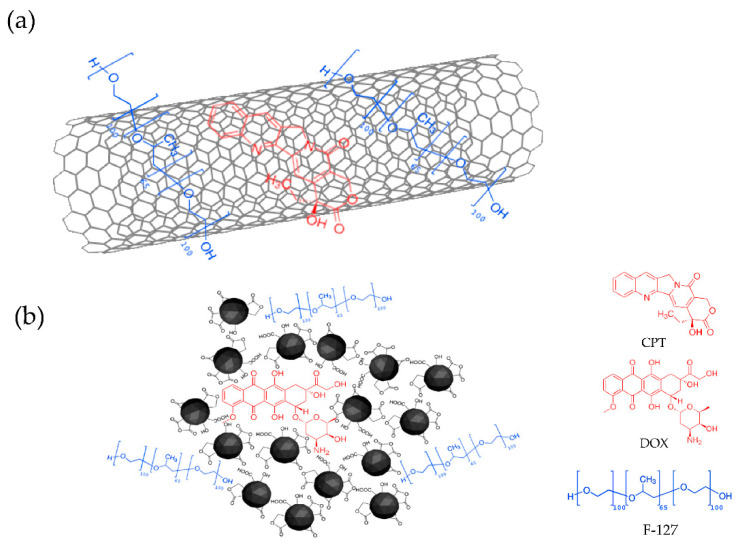
Schema of mechanism of interaction between drugs (in red) and carbon nanomaterials (in black): (**a**) CPT/CNT and (**b**) DOX/ND. (F-127 is depicted in blue).

Due to their sp^2^ carbon structure and inherent hydrophobic nature of carbon nanomaterials, all of them (except ND) are capable of establishing noncovalent π–π stacking interactions for the formation of anticancer DOX and CPT complexes. As for the hydrophobic drug CPT (Figure 7a), the more hydrophobic the carbon nanomaterial is, the more C sp^2^ domains has and the more efficient is the loading of CPT, through strong π–π interactions, which explains the results shown in Figure 6. Thus, the highest improvement in CPT anticancer activity compared to the free drug was observed for RGO and CNT nanocarriers.

On the other hand, because of its high surface free energy, ND rarely exist as a single particle, and usually form clusters of tens to hundreds of nanometers, even when they are dispersed in a solution by strong ultrasonication. Drug molecules can be assembled on the surface of ND clusters or in the nanoscale pores inside the ND clusters (Figure 7b) by noncovalent interactions [77,85]. The highest improvement in DOX activity compared to that of the free drug was observed for ND. However, ND were not efficient in loading the more hydrophobic drug, CPT. Results for higher ND concentration up to 20 µg·mL^−1^ shown in Figure S8 show that the efficiency was worse than those at 0.6 µg·mL^−1^, probably due to ND aggregation forming higher size clusters, which offer less surface area for drug loading and more difficulty to enter the cells.

Carbon nanomaterials display unique physicochemical properties making them potentially useful for bioapplications and competitive when compared to micelles, polymeric nanoparticles, dendrimers, and liposomes, to name a few. Thus, they offer high surface area for multiple drug adsorption through π–π stacking interactions and, as for ND, drugs bound to the abundant functional groups on their surface show enhanced chemotherapeutic efficacy. Much research activity has been devoted to perform in vivo experiments, either by systemic administration and localized drug delivery strategies [86]. Remarkably, carbon nanomaterials have also received much attention in imaging and diagnostics. Thus, due to their strong absorption in the IR or NIR regions, they can be used in cancer photothermal therapy (PTT). Also, they are useful in fluorescence [87,88] and photoacoustic imaging (PAI) [89,90,91]. Intrinsic carbon nanomaterial Raman vibrations allow monitoring their in vivo distribution and metabolism [32,33]. ND presenting nitrogen-vacancy centers have intrinsic fluorescence properties, and therefore are interesting tools for imaging and diagnostics [75]. Finally, carbon-based nanomaterials are emerging as potential candidates for the development of synthetic scaffolds in tissue engineering [92,93,94,95].

Long-term fate of carbon nanomaterials has been the subject of much concern and the origin of much skepticism surrounding their in vivo applications, as are presumed to be biopersistent. Despite discrepancies in findings on the clearance mechanism, majority of the studies have suggested that increasing the degree of functionalization enhanced renal clearance, while lower functionalization promoted RES accumulation (i.e., liver and spleen) [96]. Several groups have reported that carbon-based nanomaterials are susceptible to biodegradation as a result of the key role played by the immune system [97].

## 5. Conclusions

Cytotoxicity evaluation after 24 h and 72 h of incubation with various carbon nanomaterials shows differential effects on Caco-2 and MCF-7 cells. Cell viability followed the order CNP < CNH < RGO < CNT < GO < ND, being more pronounced in the more rapidly dividing Caco-2 cells. ND and GO showed the lowest toxicity, due to the presence of oxygen functional groups on carbon nanomaterial surface, that shield the hydrophobic carbon domains. High hydrophobicity, together with the morphology containing sharp conical ends in CNH and sharp edges in CNP would account for the high cell viability decrease, enhanced ROS level and apoptosis/necrosis. Remarkable high ROS levels were obtained for CNP, more pronounced on Caco-2 cells.

There is a lack of ROS generation from both cell lines after incubation with ND, as well as the lowest apoptosis values, which further supports that ND provide the lowest toxicity among the carbon nanomaterials tested here, which make them an ideal carrier for designing drug delivery systems. ND form clusters of tens to hundreds of nanometers, wherein drugs can be loaded by interaction with their surface functional groups, and therefore ND will be much more efficient in loading hydrophilic drugs, such as DOX, which readily attach to their functional groups on their surface, rather than hydrophobic drugs. In contrast, CNT and RGO, which also have low toxicity among the hydrophobic carbon nanomaterials tested here, offer available surface area for π–π interactions with aromatic rings, leading to high CPT loading efficiency, due to the strong π–π stacking interactions formed with CPT. Remarkably, CPT is a more potent anticancer agent than DOX, so developing new drug delivery systems for CPT is of high interest.

Several obstacles must be overcome before carbon nanomaterials can be suitable for clinical use. The major challenge and current limitation in this area is still the potential long-term toxicity concerns of carbon nanomaterials. Comparative in vitro studies of cytotoxicity of carbon nanomaterial synthesized from different sources are needed as screening and risk-to-benefit assessment, together with drug loading efficiency studies, to further develop advanced multi-functional carbon nanomaterials for cancer theranostic applications.

## Figures and Tables

**Table 1:** Surface chemical analysis (at.%) of the carbon nanomaterials, obtained from XPS spectra.

At.%	CNH	CNT	CNP	RGO	GO	ND ^1^
C	92.5	94.7	89.7	82.0	50.8	80.5
O	7.5	5.3	10.3	18.0	49.2	16.5

^1^ For ND, at.% N is 3.0, calculated from the N1s peak in XPS spectra.

**Table 2:** Specific surface area of carbon nanomaterials determined by BET method.

	CNH	CNT	CNP	RGO	GO	ND
Specific surface area (m^2^·g^−1^)	438.6	305.5	701.8	344.6	103.3	331.4

## Supplementary Materials

The following are available online at https://www.mdpi.com/2079-4991/10/8/1617/s1, Figure S1: High resolution XPS spectra of carbon nanomaterials; Figure S2: Cell viability assays showing the effect of F-127 at two concentrations; Figure S3: Cell viability assays after treatment with DOX and CPT, in the presence and absence of F-127; Figure S4: Cell viability assays after treatment with carbon nanomaterials at 0.6 µg·mL^−1^; Figure S5: Cell viability assays after treatment with carbon nanomaterials at 3.0 µg·mL^−1^ on human dermal fibroblast cells; Figure S6: Cell death study comparing different hydrophobic carbon nanomaterials; Figure S7: Cell cycle analysis comparing different hydrophobic carbon nanomaterials; Figure S8: Cell viability assays after treatment with ND at several concentrations, DOX and CPT.
