# Supplementary material for: Toxicity of Carbon Nanomaterials and Their Potential Application as Drug Delivery Systems: In Vitro Studies in Caco-2 and MCF-7 Cell Lines
Source: Nanomaterials (Basel). 2020 Aug 18;10(8):1617. doi: 10.3390/nano10081617 (PMC7466705; doi:10.3390/nano10081617)
Supplement: Supplementary file 1 [file nanomaterials-10-01617-s001.pdf]

nanomaterials

Supplementary Information

Toxicity of Carbon Nanomaterials and Their Potential Application as Drug Delivery Systems: In Vitro Studies in Caco-2 and MCF-7 Cell Lines

Rosa Garriga [1,*], Tania Herrero-Continente [2], Miguel Palos [3], Vicente L. Cebolla [4], Jesús Osada [2,5], Edgar Muñoz [4] and María Jesús Rodríguez-Yoldi [3,5,*]

1. Departamento de Química Física, Universidad de Zaragoza, 50009 Zaragoza, Spain
2. Departamento de Bioquímica y Biología Molecular, Universidad de Zaragoza, 50013 Zaragoza, Spain; taniaherrero1992@gmail.com (T.H.-C.); josada@unizar.es (J.O.)
3. Departamento de Farmacología y Fisiología, Universidad de Zaragoza, 50013 Zaragoza, Spain; mpalosmarg@gmail.com
4. Instituto de Carboquímica ICB-CSIC, Miguel Luesma Castán 4, 50018 Zaragoza, Spain; vcebolla@icb.csic.es (V.L.C.); edgar@icb.csic.es (E.M.)
5. CIBEROBN (ISCIII), IIS Aragón, IA2, 50009 Zaragoza, Spain

* Correspondence: rosa@unizar.es (R.G.); mjrodyol@unizar.es (M.J.R-Y.); Tel.: +34-976-762294 (R.G.); +34-976-761649 (M.J.R-Y.)

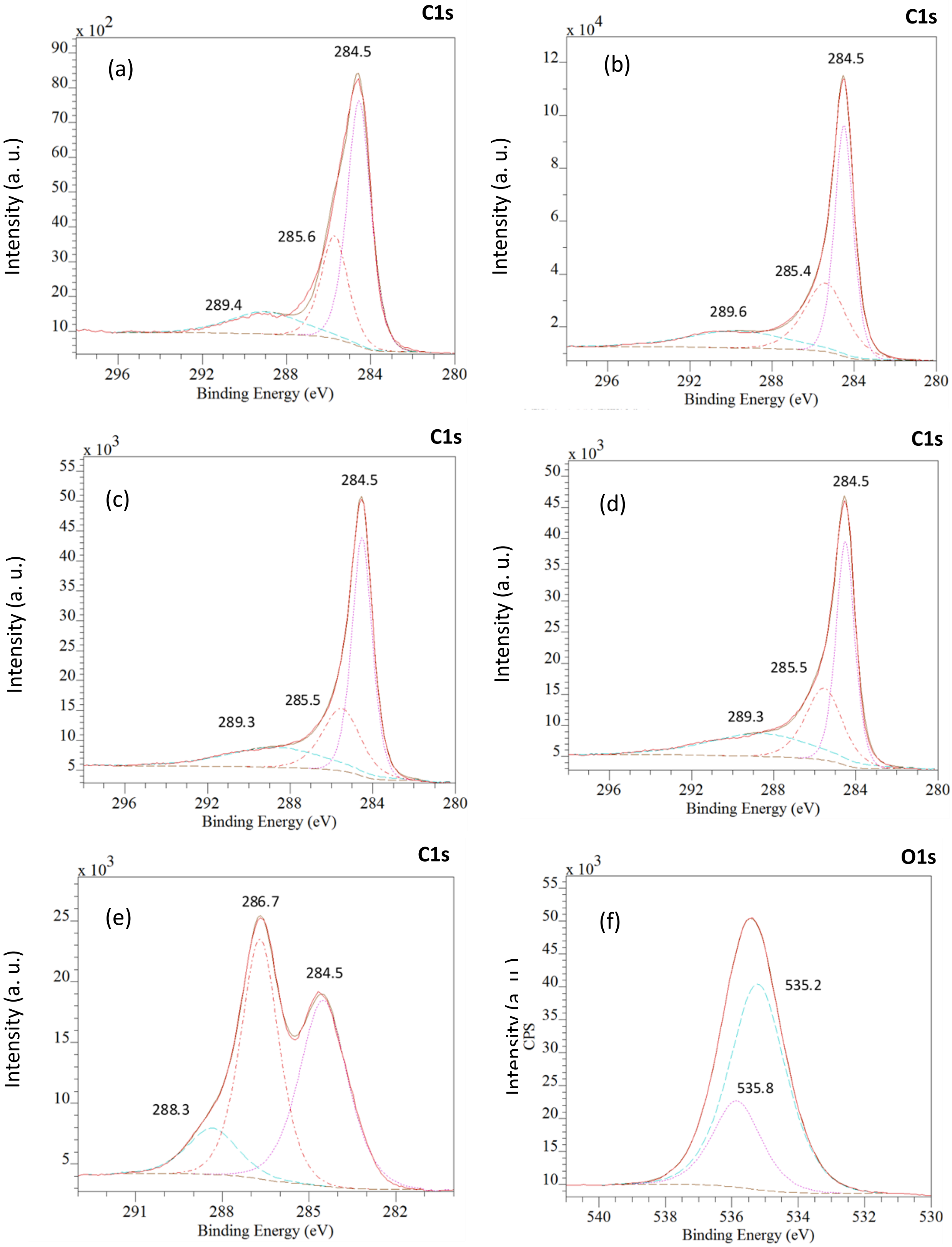

C1s
(a)
x 10^2
Intensity (a. u.)
284.5
285.6
289.4
Binding Energy (eV)
C1s
(b)
x 10^4
Intensity (a. u.)
284.5
285.4
289.6
Binding Energy (eV)
C1s
(c)
x 10^3
Intensity (a. u.)
284.5
285.5
289.3
Binding Energy (eV)
C1s
(d)
x 10^3
Intensity (a. u.)
284.5
285.5
289.3
Binding Energy (eV)
C1s
(e)
x 10^3
Intensity (a. u.)
286.7
284.5
288.3
Binding Energy (eV)
O1s
(f)
x 10^3
CPS
535.2
535.8
Binding Energy (eV)

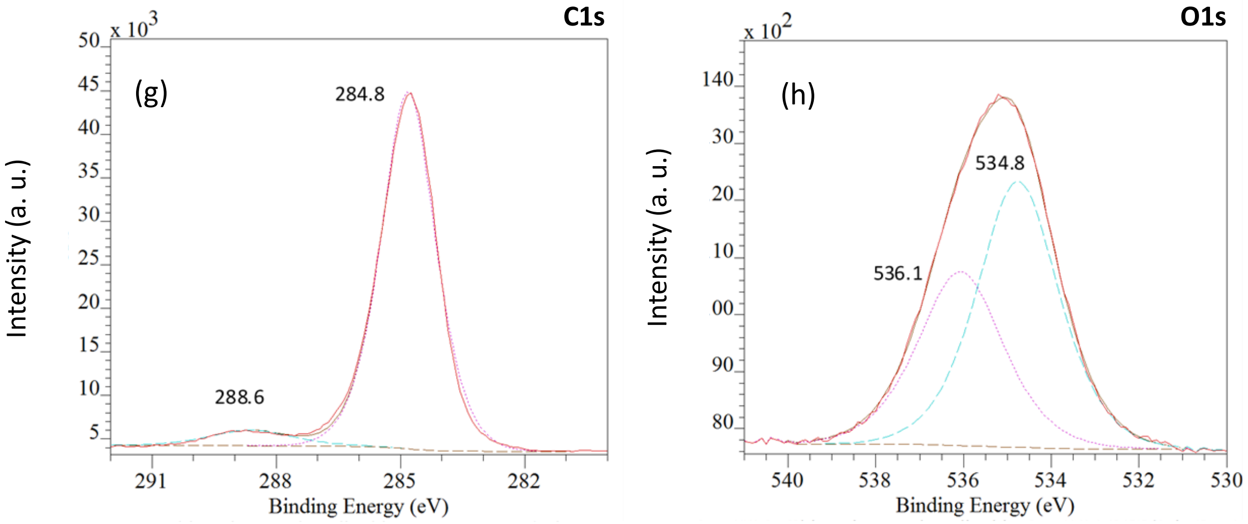

Figure S1. C1s and O1s regions in high resolution XPS spectra of carbon nanomaterials: (a) CNH, (b) CNT, (c) CNP, (d) RGO, (e, f) GO and (g, h) ND. In (a-d), C1s features at 284.5,~285.4, and ~289.5 eV are assigned to sp^2 C=C, sp^3 C-C, and π-π* transitions, respectively. In (e), C1s XPS spectra show a wide peak at 284.5 (sp^2 C=C, sp^3 C-C), at 286.7 (C-O-C, C-OH) and at 288.3 eV (C=O, COOH). In (g), C1s peaks at 284.8 and 288.6 eV correspond to sp^3 C-C diamond bonds and (C=O, COOH), respectively. Oxygen content (% at.) of these carbon nanomaterials, obtained from XPS spectra, are collected in Table 1.

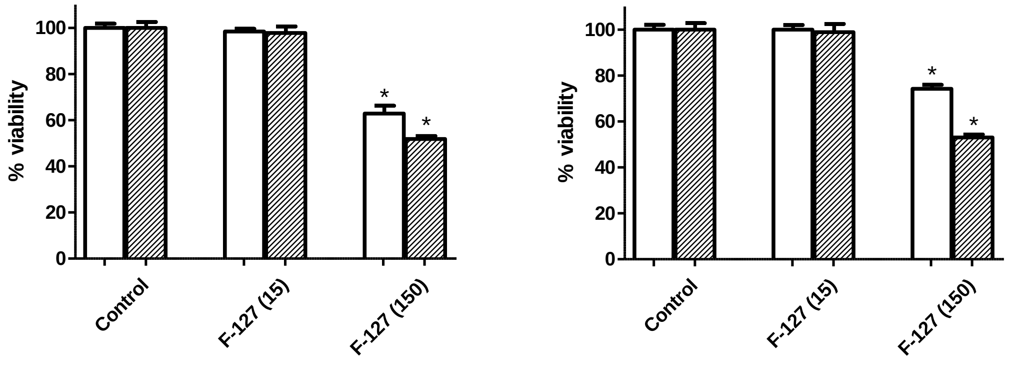

(a) (b)

Figure S2. Cell viability assays after treatment with F-127 at 15 μg·mL^{-1} and 150 μg·mL^{-1} for 24 h (white) and 72 h (striped), on (a) Caco-2 and (b) MCF-7 cells. (*represents significance at $p < 0.05$ when compared to untreated control cells).

(a)

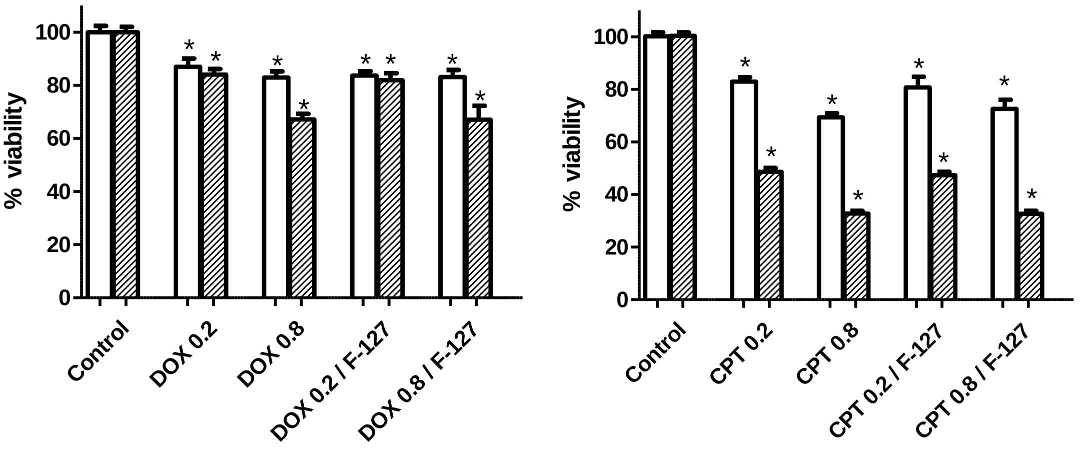

(b)

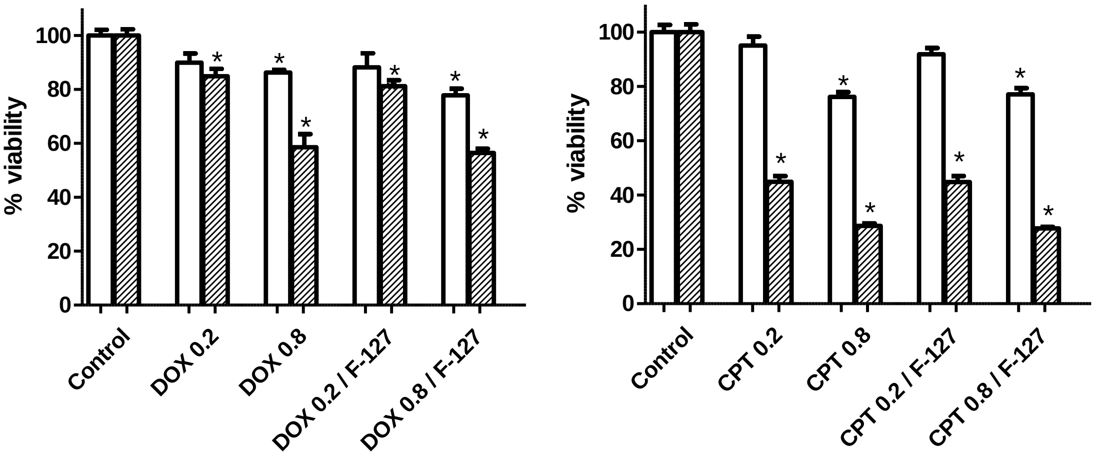

Figure S3. Cell viability assays after treatment with DOX and CPT at 0.2 $\mu g \cdot mL^{-1}$ and 0.8 $\mu g \cdot mL^{-1}$ for 24 h (white) and 72 h (striped), on (a) Caco-2 and (b) MCF-7 cells, showing no significant differences in the absence or in the presence of F-127 at 15 $\mu g \cdot mL^{-1}$. (*represents significance at $p < 0.05$ when compared to untreated control cells).

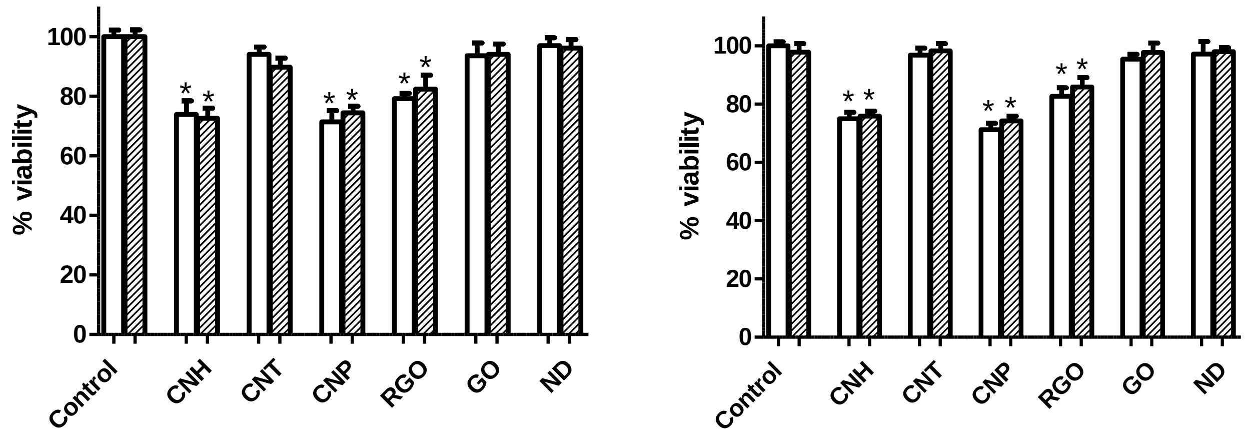

(a) (b)

Figure S4. Cell viability assays after 24 h (white) and 72 h (striped) of incubation with various carbon nanomaterials at 0.6 μg·mL^{-1} showing differential effects on (a) Caco-2 and (b) MCF-7 cells. Values that are significantly different from the control ($p < 0.05$) are denoted with asterisk (*). Untreated cells were used as control.

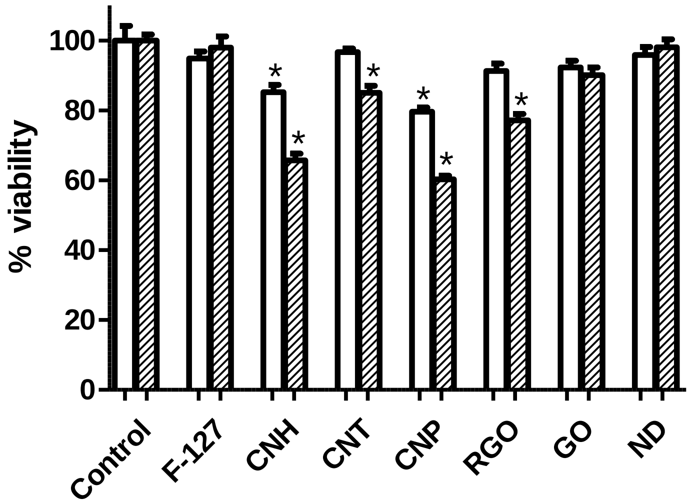

Figure S5. Cell viability assays after 24 h (white) and 72 h (striped) of incubation with various carbon nanomaterials at 3.0 μg·mL^{-1}, and also F-127 at 15 μg·mL^{-1}, showing differential effects on human dermal fibroblasts. Values that are significantly different from the control ($p < 0.05$) are denoted with asterisk (*). Untreated cells were used as control.

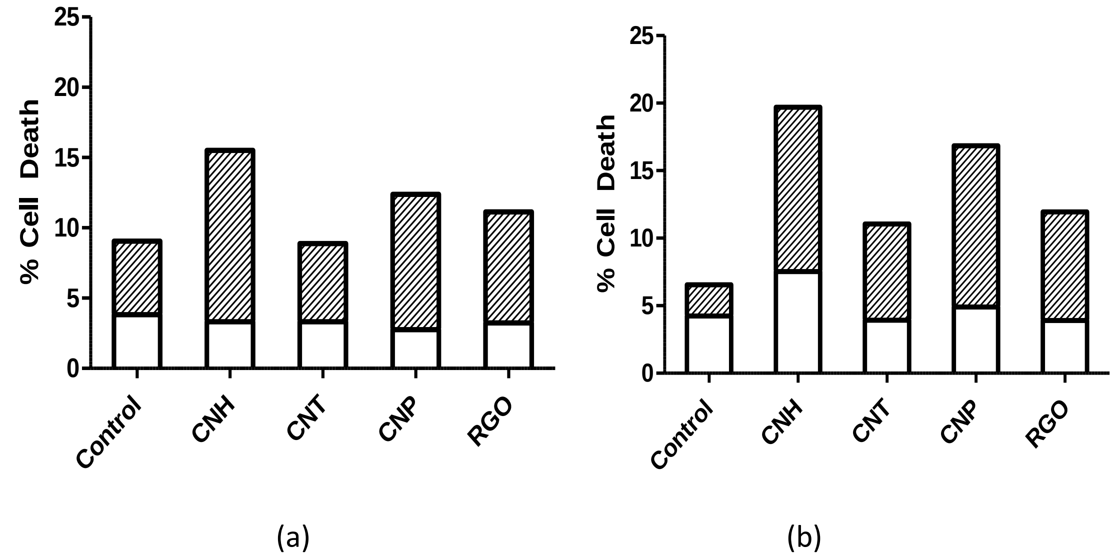

Figure S6. Bar graphs corresponding to flow cytometry analysis, quantifying the percentage of early-stage apoptotic (white), and late-stage apoptotic/necrotic (striped) cells in response to exposure for 72 h to different carbon nanomaterials at 3 $\mu g \cdot mL^{-1}$ for (a) Caco-2 and (b) MCF-7 cells. Control represents untreated cells.

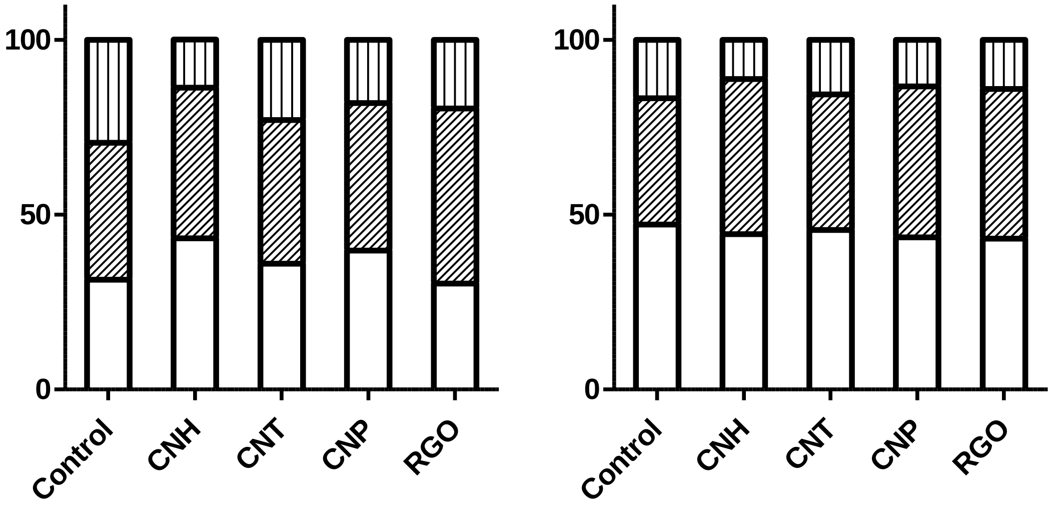

(a) (b)

Figure S7. Quantitative distribution percentages of cell cycle phases for (a) Caco-2 and (b) MCF-7, after treatment with carbon nanomaterials at 3 μg·mL^{-1} for 72 h. Control represents untreated cells. (☐ G1, ▨ S, ▥ G2).

(a)

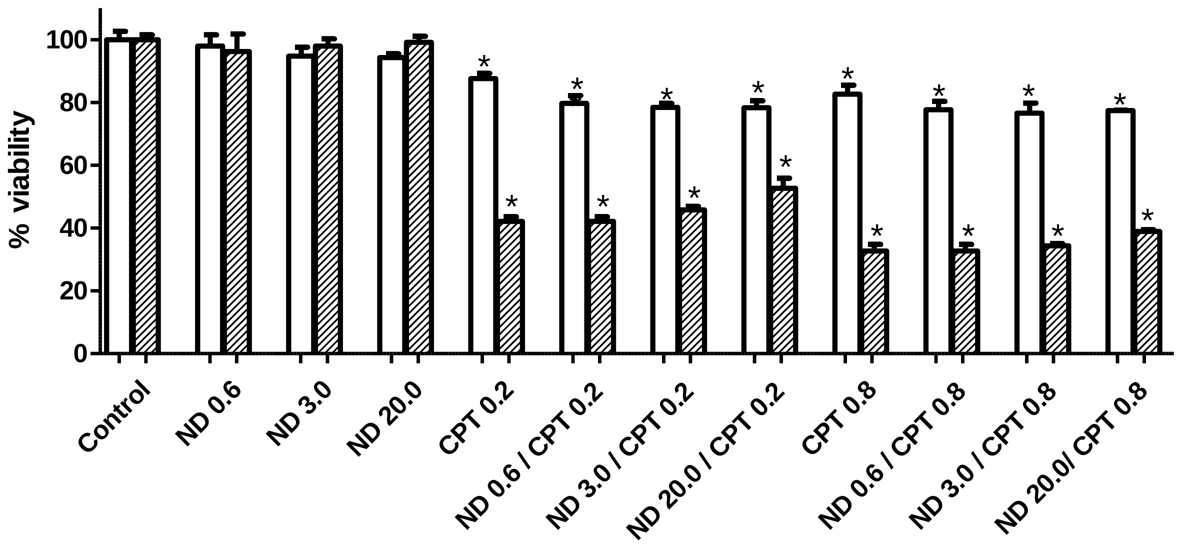

(b)

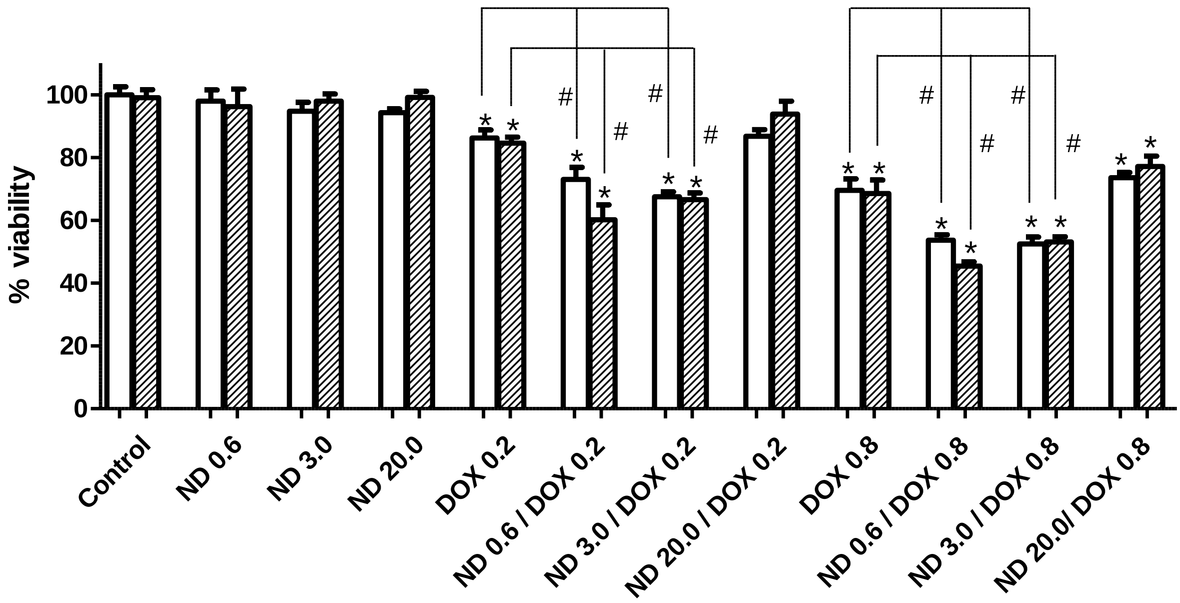

Figure S8. Cell viability assays for Caco-2 cells after 24 h (white) and 72 h (striped) of incubation with ND at 0.6, 3.0 and 20.0 μg·mL^{-1}, free drugs (a) CPT and (b) DOX, at 0.2 and 0.8 μg·mL^{-1}, and (a) CPT- and (b) DOX- loaded carbon nanomaterials. (* and # represent significance at $p < 0.05$ when compared to untreated control cells and free drug-treated cells, respectively).
